# Association between systemic immune-inflammation index and ventricular premature contractions in patients without structural heart disease

**DOI:** 10.3389/fcvm.2026.1787701

**Published:** 2026-07-29

**Authors:** Erkan Alpaslan, Sedat Taş, Ümmü Taş, Mehmet Eyüboğlu

**Affiliations:** Department of Cardiology, Faculty of Medicine, İzmir Demokrasi University, İzmir, Türkiye

**Keywords:** premature ventricular contractions, PVC burden, systemic immune-inflammation index, systemic inflammation, ventricular ectopy

## Abstract

**Background:**

Premature ventricular contractions (PVCs) are common cardiac arrhythmias that have been associated with left ventricular dysfunction, PVC-induced cardiomyopathy, and adverse cardiovascular outcomes when present in high burden. Systemic inflammation has emerged as a potential contributor to ventricular ectopy. The Systemic Immune-Inflammation Index (SII) is an easily obtainable biomarker reflecting inflammatory status. This study aimed to investigate the association between SII and both the presence and burden of PVCs.

**Methods:**

This retrospective study included 418 consecutive adult patients who underwent 24-hour Holter monitoring and complete blood count analysis. Patients were categorized according to PVC presence and further stratified by PVC burden. SII values were compared between groups and across PVC burden categories. Correlation analysis, receiver operating characteristic (ROC) analysis, and multivariable logistic regression analyses were performed to evaluate the relationship between SII and PVC burden.

**Results:**

Patients with PVCs had significantly higher SII values than controls (506.69 [354.20–674.28] vs. 463.12 [349.58–573.74], *p* = 0.012). SII increased progressively across PVC burden categories (*p* = 0.007). Spearman analysis demonstrated a weak but significant positive correlation between SII and PVC burden (rho = 0.150, *p* = 0.029). In multivariable analysis, SII remained independently associated with both PVC presence and high PVC burden. ROC analysis demonstrated a moderate discriminatory performance of SII for identifying high PVC burden (AUC = 0.696, 95% CI: 0.597–0.795, *p* < 0.001), with an optimal cut-off value of 505.6. Among the evaluated inflammatory biomarkers, SII showed the largest AUC for identifying high PVC burden, followed by NLR and PLR.

**Conclusion:**

Higher SII levels were associated with both the presence and increasing burden of PVCs. These findings support a potential relationship between systemic inflammatory status and ventricular ectopy and suggest that SII may provide complementary information when interpreted together with clinical evaluation and rhythm monitoring, rather than as a standalone marker.

## 1 Introduction

Premature ventricular contractions (PVCs) are among the most common cardiac electrical abnormalities observed both in healthy individuals and in patients with structural heart disease (1). Although PVCs are often considered benign in patients without overt structural heart disease, frequent PVCs have been associated with left ventricular dysfunction, PVC-induced cardiomyopathy, heart failure, and adverse cardiovascular outcomes (2, 3). A secondary analysis of the CHF-STAT study provided important evidence supporting the clinical relevance of frequent PVCs by showing that suppression of PVCs with amiodarone was associated with improvement in left ventricular function and confirmation of PVC-induced cardiomyopathy in a substantial proportion of patients (4). Therefore, PVCs may represent more than a simple electrophysiological finding and may be associated with complex pathophysiological processes linked to adverse cardiovascular outcomes, including heart failure.

Recent studies have increasingly highlighted the pivotal role of inflammation in the development of PVCs related cardiovascular diseases (6, 7). Among the hematologic markers obtained from routine complete blood counts, inflammatory indices have gained interest as affordable, easily available, and potentially effective indicators for predicting outcomes. The neutrophil-to-lymphocyte ratio (NLR) and the platelet-to-lymphocyte ratio (PLR) have been widely studied as possible indicators of negative cardiovascular outcomes (8). PVCs are known to be associated with increased inflamatuar markers through promoting inflammatory and structural and functional remodeling pathways of the heart following electrophysiological changes. In line with above mentioned mechanisms, Keles et al. suggested that premature ventricular complexes can lead to atrial remodeling as well as ventricular remodeling (9). Furthermore, in a recent study, Hamon et al. speculated that PVCs influence intrinsic cardiac nervous system neurons and alter cardiac repolarization and those alterations may be pivotal for arrhythmogenesis and remodeling, resulting in cardiomyopathy (10).

Emerging evidence suggests that inflammation may contribute to the development and persistence of ventricular ectopy. The MAVERIC registry demonstrated that a substantial proportion of patients presenting with frequent symptomatic PVCs and no known ischemic heart disease had evidence of myocardial inflammation on advanced multimodality imaging. These findings suggest that some apparently idiopathic PVCs may be associated with an underlying inflammatory myocardial substrate. In addition, frequent PVCs have been associated with the development of PVC-induced cardiomyopathy, highlighting the importance of identifying patients who may have an increased burden of ventricular ectopy and potential underlying myocardial abnormalities (11).

In 2014, Hu and his team published that SII, a new, comprehensive marker that includes those aferomentioned indicators (12). The Systemic immunological-Inflammation Index, derived using the formula (platelet × neutrophil)/lymphocyte, provides a single measure that reflects both inflammatory and immunological responses. Previous researches have shown that SII can predict outcomes in numerous cardiovascular diseases, including stroke, heart failure, and acute coronary syndromes (13–15). Increased SII levels demonstrated to be a better predictor of significant cardiovascular problems than either NLR or PLR in patients with coronary artery disease (16, 17). However, evidence regarding the association between SII and ventricular premature beats remains limited, particularly in patients without structural heart disease. Therefore, this study aimed to investigate the association between SII and the presence and burden of PVCs assessed by 24-hour Holter monitoring.

## 2 Material and methods

In this retrospective study, the study population consisted of 418 consecutive adult patients who underwent 24-hour Holter monitoring and had available laboratory and echocardiographic data at the cardiology outpatient clinics of our hospital between November 2020 and September 2025. Patients with known coronary artery disease, heart failure, structural heart disease, active infection, malignancy, autoimmune or chronic inflammatory disease, hematological disorders, immunosuppressive therapy, or reduced left ventricular systolic function were excluded. Reduced left ventricular systolic function was defined as LVEF <50%; therefore, all included patients had preserved LVEF (≥50%). LVEF was nevertheless recorded and included in the multivariable models to account for residual variation within the preserved LVEF range. The control group consisted of patients who fulfilled the study eligibility criteria, had available laboratory and echocardiographic data, and had no PVCs detected on 24-hour Holter monitoring.

This study was reported in accordance with the TRIPOD reporting guidance for prediction model validation studies where applicable. The present analysis evaluated the discriminatory and predictive performance of SII for PVC presence and high PVC burden. Because the cohort was restricted to consecutive eligible patients with available 24-hour Holter monitoring, complete blood count, and echocardiographic data during the study period, complete-case analysis was performed.

The study protocol was approved by the İzmir Demokrasi University Buca Seyfi Demirsoy Training and Research Hospital Ethics Committee (Date: 25.09.2025, Approval No: 2025/508) and conducted in accordance with the Declaration of Helsinki. Permission for archival data review was obtained from the hospital administration. Patients with heart failure, coronary artery disease, and structural heart disease were excluded to minimize potential confounding from conditions known to be independently associated with both systemic inflammation and ventricular arrhythmias. Participants were subsequently divided into two groups according to 24-hour Holter monitoring results: patients with PVCs (Group 1) and patients without PVCs (Group 2).

Patients with PVCs were categorized into three predefined groups according to PVC burden: low burden (<500 PVCs/24 h), intermediate burden (500–10,000 PVCs/24 h), and high burden (≥10,000 PVCs/24 h). The threshold of ≥10,000 PVCs/24 h was selected because it has been associated with an increased risk of PVC-induced cardiomyopathy in previous studies (1).

We collected demographic features, comorbidities (hypertension, diabetes mellitus), and hematological parameters of the patients. We calculated the Systemic Immune-Inflammation Index (SII) using the following formula: SII = (platelet count × neutrophil count)/lymphocyte count.

### 2.1 Statistical analysis

The statistical analyses were conducted using IBM SPSS Statistics version 27.0. The Kolmogorov–Smirnov test was used to assess normality. Continuous variables were presented as mean ± standard deviation or median (interquartile range), as appropriate, while categorical variables were presented as counts and percentages. Independent samples *t*-test was used to compare normally distributed continuous variables, whereas the Mann–Whitney *U*-test was used for non-normally distributed variables. Chi-square test was used for categorical variables. The three PVC burden subgroups were compared using one-way ANOVA, followed by *post hoc* pairwise comparisons with Bonferroni correction when appropriate. The diagnostic performance of SII for predicting high PVC burden was assessed using receiver operating characteristic (ROC) analysis. Multivariable logistic regression analysis was performed to identify independent predictors of PVC presence and high PVC burden. Spearman's rank correlation analysis was used to evaluate the relationship between SII and total PVC count among patients with PVCs. Neutrophil-to-lymphocyte ratio (NLR) and platelet-to-lymphocyte ratio (PLR) were calculated from complete blood count parameters. Comparative ROC analyses were performed to evaluate the discriminatory performance of SII, NLR, and PLR for identifying high PVC burden (≥10,000 PVCs/24 h). A *p*-value <0.05 was considered statistically significant.

## 3 Results

The mean age was 49.12 ± 11.77 years in the PVC group and 47.00 ± 12.93 years in the control group, with no statistically significant difference between the groups (*p* = 0.080). The prevalence of hypertension was 25.7% (*n* = 55) in the PVC group and 19.1% (*n* = 39) in the control group (*p* = 0.100). Diabetes mellitus was present in 5.6% (*n* = 12) of patients in the PVC group and 6.8% (*n* = 14) of those in the control group (*p* = 0.500). Female sex was less frequent in the PVC group than in the control group [50.4% (*n* = 108) vs. 60.7% (*n* = 124), *p* = 0.039]. Left ventricular ejection fraction was preserved and similar between the PVC and control groups (62.78 ± 2.46% vs. 63.03 ± 2.39%, *p* = 0.295). No significant differences were observed in white blood cell count, neutrophil count, platelet count, hemoglobin level, or monocyte count between the groups (all *p* > 0.05). Patients with PVCs had lower lymphocyte counts and higher SII values compared with controls [2.17 (1.80–2.70) vs. 2.39 (2.09–2.87), *p* < 0.001; and 506.69 (354.20–674.28) vs. 463.12 (349.58–573.74), *p* = 0.012, respectively]. The results are shown in Table 1.

**Table 1:** Baseline demographic, clinical, echocardiographic, and laboratory characteristics of patients With and without premature ventricular contractions.

Variable	PVC Group	Control Group	*p*-value
	(*n* = 214)	(*n* = 204)	
Age, years	49.12 ± 11.77	47.00 ± 12.93	0.080
Female sex, *n* (%)	108 (50.4)	124 (60.7)	**0**.**039**
Hypertension, *n* (%)	55 (25.7)	39 (19.1)	0.100
Diabetes mellitus, *n* (%)	12 (5.6)	14 (6.8)	0.500
LVEF, %	62.78 ± 2.46	63.03 ± 2.39	0.295
WBC, ×10^9^/L	7.51 ± 1.47	7.49 ± 1.52	0.871
Neutrophil, ×10^9^/L	4.48 ± 1.20	4.32 ± 1.15	0.149
Platelet, ×10^9^/L	262.76 ± 82.57	263.37 ± 60.42	0.932
Hemoglobin, g/dL	13.47 ± 1.62	13.23 ± 1.69	0.126
Lymphocyte, ×10^9^/L, median (Q1–Q3)	2.17 (1.80–2.70)	2.39 (2.09–2.87)	**<0**.**001**
Monocyte, ×10^9^/L, median (Q1–Q3)	0.60 (0.46–0.73)	0.59 (0.48–0.70)	0.496
SII, median (Q1–Q3)	506.69 (354.20–674.28)	463.12 (349.58–573.74)	**0**.**012**

PVC, premature ventricular contraction; SII, systemic immune-inflammation index; LVEF, left ventricular ejection fraction; WBC, white blood cell count. Continuous variables are presented as mean ± standard deviation or median (Q1–Q3), as appropriate.

Bold values indicate statistical significance (*p* < 0.05).

Among the 214 patients with PVCs, 86 patients (40.2%) had low PVC burden (<500 PVCs/24 h), 96 patients (44.9%) had intermediate PVC burden (500–10,000 PVCs/24 h), and 32 patients (15.0%) had high PVC burden (≥10,000 PVCs/24 h). When considered within the overall study population of 418 patients, the corresponding proportions were 20.6%, 23.0%, and 7.7%, respectively. These proportions should be interpreted in the context of the selected study population, which included only patients with available Holter, laboratory, and echocardiographic data and excluded patients with structural heart disease, coronary artery disease, heart failure, active inflammatory conditions, or reduced left ventricular systolic function.

### 3.1 Exploratory analysis of PVC prevalence across SII tertiles in the overall study population

As an exploratory analysis, SII tertiles were calculated in the overall study population, including both patients with PVCs and controls without PVCs (*n* = 418). In this analysis, the outcome was the presence of any PVC detected on 24-hour Holter monitoring, rather than high PVC burden (≥10,000 PVCs/24 h). The prevalence of PVCs differed significantly across SII tertiles (*χ*^2^ = 8.137, *p* = 0.017). PVC prevalence was 49.6% in the lowest tertile, 43.6% in the intermediate tertile, and 60.4% in the highest tertile. Because the distribution was not strictly stepwise across tertiles, this exploratory finding should be interpreted cautiously. Nevertheless, the highest SII tertile showed the greatest prevalence of PVCs, supporting a possible association between higher systemic inflammatory status and the presence of ventricular ectopy.

### 3.2 Comparison of SII across PVC burden categories among patients with PVCs

**Figure 1:**
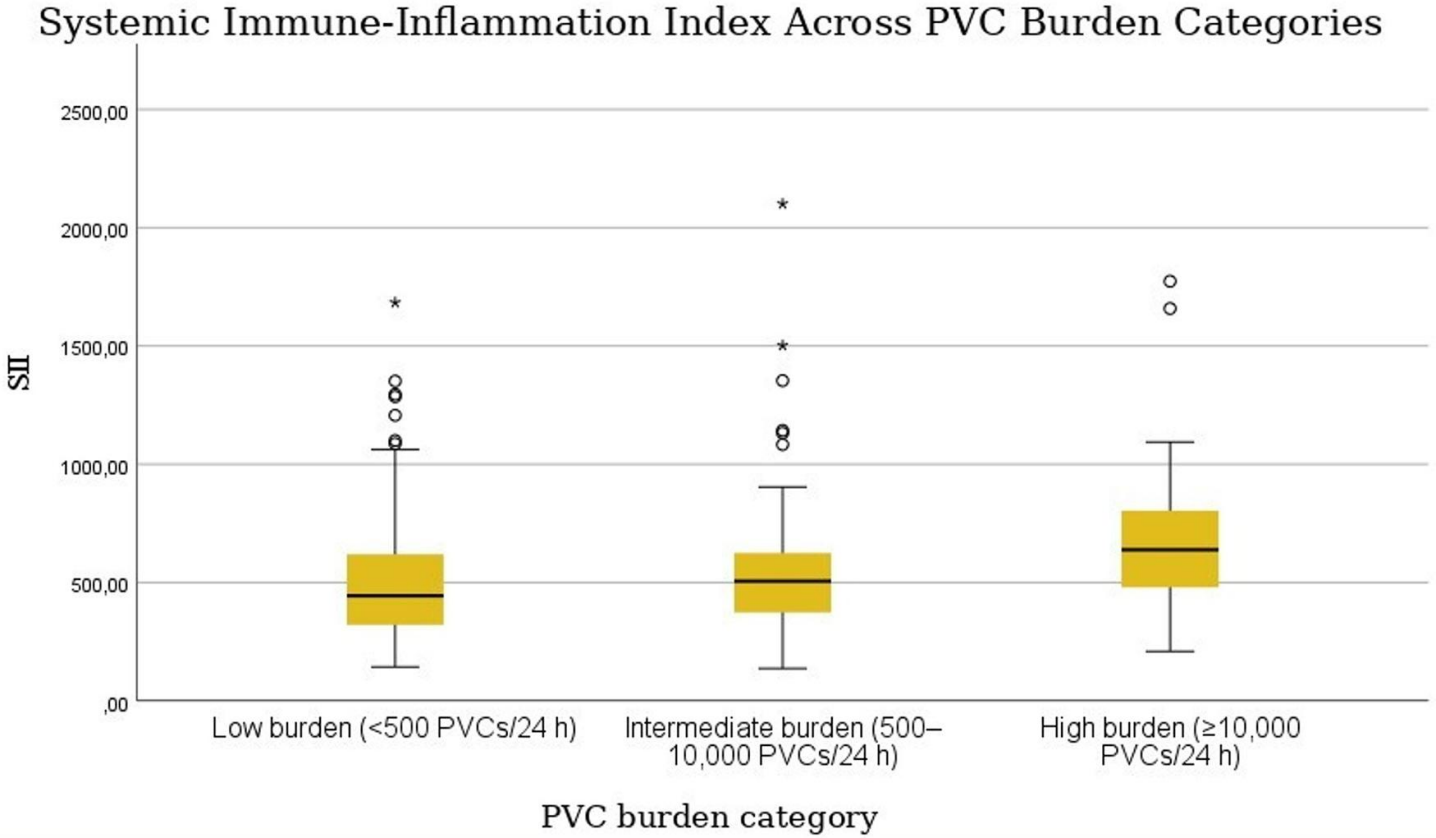
Boxplot showing systemic immune-inflammation index (SII) values across predefined premature ventricular contraction (PVC) burden categories. SII values differed significantly across groups (ANOVA, *p* = 0.007). The boxes represent the interquartile range, horizontal lines indicate medians, whiskers show the non-outlier range, circles indicate outliers, and asterisks indicate extreme outliers.

Among patients with PVCs only (*n* = 214), SII values were compared across predefined PVC burden categories: low burden (<500 PVCs/24 h, *n* = 86), intermediate burden (500–10,000 PVCs/24 h, *n* = 96), and high burden (≥10,000 PVCs/24 h, *n* = 32). SII values differed significantly across these groups (ANOVA, *p* = 0.007), with the highest values observed in patients with high PVC burden (Figure 1). Pairwise comparisons with Bonferroni correction showed significant differences between the low- and high-burden groups (adjusted *p* = 0.009) and between the intermediate- and high-burden groups (adjusted *p* = 0.033), whereas no significant difference was observed between the low- and intermediate-burden groups (adjusted *p* = 0.636).

### 3.3 Correlation analysis between SII and PVC burden

**Figure 2:**
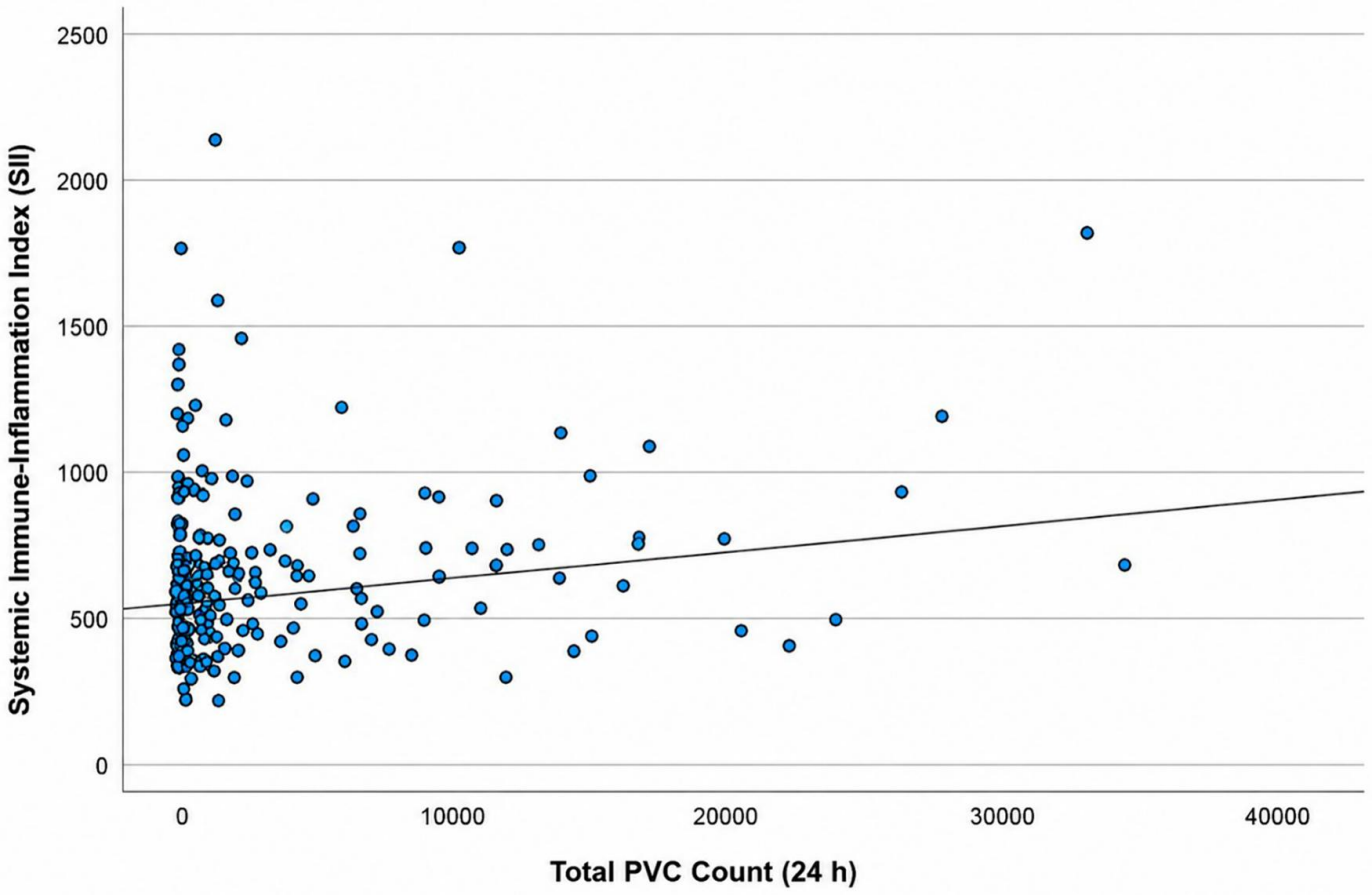
Scatter plot showing the relationship between systemic immune-inflammation index (SII) and total PVC count. A weak but significant positive correlation was observed (Spearman rho = 0.150, *p* = 0.029).

A Spearman correlation analysis was performed among patients with PVCs to evaluate the relationship between SII and PVC burden. The analysis demonstrated a weak but statistically significant positive correlation between SII and PVC count (rho = 0.150, *p* = 0.029), indicating that higher SII levels were associated with a greater PVC burden. The relationship is illustrated in Figure 2.

### 3.4 Subgroup analysis: diagnostic performance of SII for predicting high PVC burden

We evaluated the discriminative ability of the systemic immune-inflammation index (SII) for identifying patients with a high burden of premature ventricular contractions (PVCs). High PVC burden was defined as ≥10,000 PVCs/24 h. In the revised ROC analysis, patients with high PVC burden were classified as the positive group (*n* = 32), whereas all remaining participants, including those without PVCs and those with lower PVC burdens, were included in the negative group (*n* = 386).

**Figure 3:**
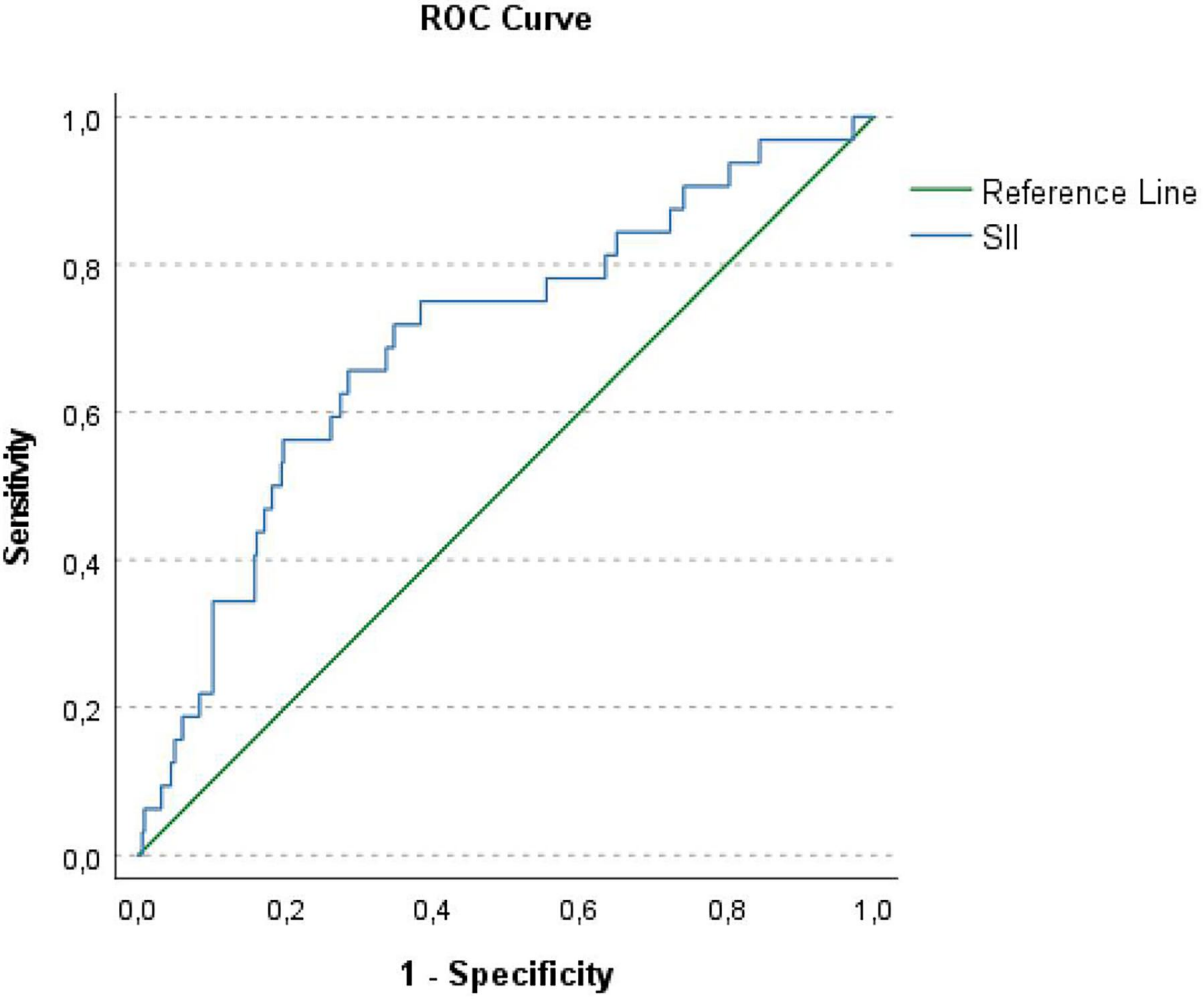
ROC curve of the systemic immune-inflammation index (SII) for predicting high PVC burden (≥10,000 PVCs/24 h). The area under the curve was 0.696 (95% CI: 0.597–0.795, *p* < 0.001).

ROC curve analysis demonstrated that SII had a significant discriminatory ability for identifying patients with high PVC burden, with an area under the curve (AUC) of 0.696 (95% CI: 0.597–0.795, *p* < 0.001). The optimal SII cut-off value determined by the Youden index was 505.6, yielding a sensitivity of 50.9% and a specificity of 66.2% (J = 0.171). At this threshold, the positive predictive value was 11.1% and the negative predictive value was 94.2%. The results are presented in Table 2 and Figure 3.

**Table 2:** ROC analysis and predictive performance of SII for identifying high PVC burden (≥10,000 PVCs/24 h).

Parameter	Value
Area under the curve (AUC)	0.696
95% confidence interval	0.597–0.795
*p*-value	<0.001
Optimal SII cut-off	505.6
Sensitivity	50.9%
Specificity	66.2%
Positive predictive value	11.1%
Negative predictive value	94.2%
Youden Index (J)	0.171

High PVC burden was defined as ≥10,000 PVCs/24 h. The remaining participants, including patients without PVCs and those with lower PVC burdens, served as the negative group. AUC, area under the curve; CI, confidence interval; PVC, premature ventricular contraction; ROC, receiver operating characteristic; SII, systemic immune-inflammation index.

### 3.5 Additional inflammatory biomarker analysis

In addition to SII, neutrophil-to-lymphocyte ratio (NLR) and platelet-to-lymphocyte ratio (PLR) were evaluated. Patients with PVCs had significantly higher NLR and PLR values than the control group. Median NLR was 1.93 (IQR: 1.50–2.65) in the PVC group and 1.74 (IQR: 1.39–2.11) in the control group (*p* < 0.001). Median PLR values were 117.15 (IQR: 86.23–152.82) and 109.67 (IQR: 88.28–128.30), respectively (*p* = 0.028). In ROC analyses, SII showed the largest AUC for identifying patients with high PVC burden (AUC = 0.696, 95% CI: 0.597–0.795, *p* < 0.001), followed by NLR (AUC = 0.647, 95% CI: 0.546–0.748, *p* = 0.006) and PLR (AUC = 0.607, 95% CI: 0.490–0.723, *p* = 0.045) (Table 3).

**Table 3:** ROC analysis of inflammatory biomarkers for identifying high PVC burden.

Variable	AUC (95% CI)	*p* value
SII	0.696 (0.597–0.795)	<0.001
NLR	0.647 (0.546–0.748)	0.006
PLR	0.607 (0.490–0.723)	0.045

AUC, area under the curve; CI, confidence interval; NLR, neutrophil-to-lymphocyte ratio; PLR, platelet-to-lymphocyte ratio; ROC, receiver operating characteristic; SII, systemic immune-inflammation index. *P* values indicate the statistical significance of each individual AUC. No formal pairwise comparison between ROC curves was performed.

### 3.6 Multivariate logistic regression analysis for predictors of PVCs

We performed multivariate logistic regression analysis to identify factors independently associated with the presence of PVCs. The dependent variable was coded as PVC presence = 1 and control/no PVC = 0. Variables included gender, age, hypertension, diabetes mellitus, Systemic Immune-Inflammation Index (SII), white blood cell count (WBC), red blood cell count (RBC), monocyte count, hemoglobin, and left ventricular ejection fraction (LVEF). In this model, SII remained independently associated with PVC presence (B = 0.002, Wald = 13.841, *p* < 0.001, OR = 1.002, 95% CI: 1.001–1.003). In this selected cohort with preserved LVEF, LVEF was not independently associated with PVC presence (B = −0.047, *p* = 0.268, OR = 0.954, 95% CI: 0.878–1.037). SII remained independently associated with PVC presence after adjustment for demographic, clinical, hematologic, and echocardiographic variables, including LVEF. Other variables, including age, gender, hypertension, diabetes mellitus, WBC, RBC, monocyte count, and hemoglobin, were not independently associated with PVC presence (all *p* > 0.05), although hemoglobin showed a borderline association (*p* = 0.053).

These findings indicate an independent association between systemic inflammatory status and PVC presence after adjustment for demographic, clinical, hematologic, and echocardiographic variables. The results are presented in Table 4.

**Table 4:** Multivariable logistic regression analysis for predictors of PVC presence.

Variable	B	Wald	*p* value	OR (Exp[B])	95% CI for OR
Age	0.007	0.475	0.491	1.007	0.988–1.026
Female sex	−0.192	0.634	0.426	0.825	0.515–1.324
Hypertension	0.353	1.573	0.210	1.424	0.820–2.474
Diabetes mellitus	−0.535	1.405	0.236	0.586	0.242–1.418
SII	0.002	13.841	<0.001	1.002	1.001–1.003
WBC	−0.144	2.645	0.104	0.866	0.728–1.030
Monocyte count	1.062	2.116	0.146	2.891	0.691–12.085
Hemoglobin	0.145	3.733	0.053	1.156	0.998–1.338
RBC	−0.058	0.590	0.442	0.944	0.815–1.094
LVEF	−0.047	1.226	0.268	0.954	0.878–1.037

PVC, premature ventricular contraction; SII, systemic immune-inflammation index; LVEF, left ventricular ejection fraction; WBC, white blood cell count; RBC, red blood cell count; OR, odds ratio; CI, confidence interval. The dependent variable was PVC presence, coded as 1 for patients with PVCs and 0 for controls.

### 3.7 Multivariate logistic regression analysis for predictors of high PVC burden (≥10,000 PVCs/24 h): subgroup analysis

We performed a multivariate logistic regression analysis to identify factors independently associated with a high PVC burden (≥10,000 PVCs/24 h). In the multivariable model, SII remained the only independent predictor of high PVC burden (B = 0.002, Wald = 9.304, *p* = 0.002, OR = 1.002, 95% CI: 1.001–1.003). This finding suggests that increasing levels of systemic inflammation are independently associated with a greater likelihood of frequent ventricular ectopy.

Male sex showed a borderline association with high PVC burden (B = 0.861, *p* = 0.058, OR = 2.366, 95% CI: 0.970–5.773), but did not reach statistical significance. Similarly, age, hypertension, diabetes mellitus, left ventricular ejection fraction (LVEF), white blood cell count (WBC), monocyte count, hemoglobin level, and red blood cell (RBC) count were not independently associated with high PVC burden (all *p* > 0.05). The results are presented in Table 5.

**Table 5:** Multivariable logistic regression analysis for predictors of high PVC burden (≥10,000 PVCs/24 h).

Variable	B	Wald	*p*-value	OR (Exp[B])	95% CI for OR
Age	0.018	0.945	0.331	1.018	0.982–1.056
Sex	0.861	3.584	0.058	2.366	0.970–5.773
Hypertension	0.277	0.299	0.584	1.319	0.489–3.552
Diabetes mellitus	0.502	0.349	0.555	1.652	0.313–8.730
SII	0.002	9.304	**0**.**002**	1.002	1.001–1.003
LVEF	−0.069	0.791	0.374	0.933	0.800–1.087
WBC	0.169	1.025	0.311	1.185	0.853–1.644
Monocyte count	−0.433	0.109	0.742	0.649	0.049–8.504
Hemoglobin	0.033	0.053	0.817	1.033	0.782–1.366
RBC	−0.038	0.079	0.778	0.963	0.741–1.252

PVC, premature ventricular contraction; SII, systemic immune-inflammation index; LVEF, left ventricular ejection fraction; WBC, white blood cell count; RBC, red blood cell count; OR, odds ratio; CI, confidence interval.

Bold values indicate statistical significance (*p* < 0.05).

## 4 Discussion

The main findings of the present study were as follows: (i) patients with PVCs had higher SII levels than those without PVCs; (ii) SII levels were higher across increasing PVC burden categories, with the highest values observed in patients with a high PVC burden (≥10,000 PVCs/24 h); (iii) SII was independently associated with PVC presence in multivariable analysis; (iv) SII was also independently associated with high PVC burden in the subgroup analysis; and (v) SII demonstrated a significant but moderate ability to discriminate patients with high PVC burden in ROC analysis performed across the entire study population. These findings suggest that systemic inflammatory status may be associated with both the presence and burden of ventricular ectopy.

Inflammation has been proposed to contribute to ventricular ectopy through cytokine-mediated pathways, oxidative stress, and autonomic instability, leading to electrical and structural remodeling of the myocardium (18). Previous studies have shown that patients with frequent premature ventricular contractions exhibit higher levels of inflammatory markers, including C-reactive protein (CRP), hematologic parameters, the neutrophil-to-lymphocyte ratio (NLR), and the platelet-to-lymphocyte ratio (PLR) (19–21). However, the interaction between inflammation and PVCs may be more complex than a unidirectional causal pathway. While systemic inflammation may facilitate the development or persistence of ventricular ectopy, recent experimental evidence suggests that frequent PVCs themselves may activate local inflammatory signaling within cardiomyocytes and promote profibrotic myocardial remodeling in models of PVC-induced cardiomyopathy (6). This bidirectional interaction may create a self-amplifying process in which inflammatory activation promotes ventricular ectopy, while frequent PVCs further enhance local inflammatory and fibrotic pathways within the myocardium. In this context, the significantly higher SII, NLR, and PLR values observed in patients with PVCs in the present study may reflect, at least in part, the complex interplay between systemic inflammatory status and ventricular ectopic activity. Since SII integrates neutrophil, lymphocyte, and platelet counts into a single index, it may provide a broader reflection of systemic inflammatory status than individual hematologic parameters alone (22).

In our study, when patients were categorized by PVCs burden, SII levels showed a stepwise increase across groups, with the highest values observed in those with ≥10,000 PVCs/24 h. This pattern is consistent with previous evidence suggesting a role of inflammation in ventricular ectopy. Chen et al. demonstrated that elevated hs-CRP levels were associated with an increased risk of future PVC occurrence, supporting the concept that systemic inflammation may be involved in the development of ventricular ectopy (23). These findings suggest a potential association between systemic inflammation and PVC frequency. In addition, Spearman correlation analysis demonstrated a weak but statistically significant positive correlation between SII and PVC burden. Although the strength of the correlation was modest, these findings further support the association between systemic inflammatory status and ventricular ectopy severity and suggest that increasing inflammatory burden may be related to increasing PVC frequency.

The findings of the MAVERIC registry suggest that a substantial proportion of patients presenting with frequent PVCs may have underlying myocardial inflammation despite the absence of overt structural heart disease. Approximately half of the patients evaluated with advanced multimodality imaging demonstrated evidence of myocardial inflammation or myocarditis. These observations indicate that apparently idiopathic ventricular ectopy may, in some cases, be associated with a subclinical inflammatory myocardial substrate. Therefore, the association between elevated SII levels and both the presence and burden of PVCs observed in our study may reflect not only systemic inflammation but also a possible underlying inflammatory process related to ventricular ectopy. Given that SII can be readily calculated from routine blood counts, it may represent a simple and cost-effective tool for identifying patients with frequent PVCs who may benefit from further clinical evaluation (11).

Discrepant findings have been reported in the literature regarding the relationship between PVC burden and left ventricular dysfunction. While some investigators have questioned whether PVC burden alone independently predicts cardiomyopathy, others have demonstrated a significant association between frequent PVCs and impaired ventricular function (3, 5, 24). Because left ventricular systolic dysfunction may represent a potential confounder, we additionally included LVEF in our multivariable model. Notably, LVEF was not independently associated with PVC presence, whereas SII remained significantly associated after adjustment for demographic, clinical, hematologic, and echocardiographic variables. Because patients with reduced LVEF were excluded, all participants in the present study had preserved left ventricular systolic function. Therefore, our findings should not be interpreted as demonstrating that SII is associated with PVCs independently of clinically meaningful systolic dysfunction. Rather, the inclusion of LVEF in the multivariable model was intended to account for residual variation within the preserved LVEF range. In this context, SII remained significantly associated with PVC presence after adjustment for LVEF and other demographic, clinical, hematologic, and echocardiographic variables. These findings suggest that systemic inflammatory status may be associated with ventricular ectopy even among patients without overt structural heart disease or reduced systolic function.

In the ROC analysis including the entire study population, SII demonstrated a significant but moderate ability to discriminate patients with a high PVC burden from all other participants, including those without PVCs and those with lower PVC burdens (AUC = 0.696, 95% CI: 0.597–0.795, *p* < 0.001). The optimal SII cut-off value was 505.6, yielding a sensitivity of 50.9% and a specificity of 66.2%. Among the evaluated inflammatory indices, SII yielded the largest AUC in descriptive ROC assessment; however, formal pairwise comparisons between ROC curves were not performed. Given the modest AUC, moderate sensitivity and specificity, and low positive predictive value, SII alone has limited standalone clinical utility and should not be interpreted as a diagnostic or decision-making marker for identifying high PVC burden. Rather, SII may serve as an adjunctive inflammatory biomarker that provides complementary information when interpreted together with clinical evaluation and rhythm monitoring. These findings are consistent with previous studies reporting associations between inflammation-based biomarkers, ventricular ectopy, and adverse cardiac outcomes (25, 26).

In the multivariable analysis, sex was not independently associated with PVC presence in the present cohort. The effects of sex differences on ventricular arrhythmias remain limited and inconsistent in the literature. Some studies have reported that women have a lower incidence of cumulative ventricular arrhythmias in both ischemic and non-ischemic settings. Moreover, among patients without structural heart disease, men have been reported to have a higher incidence of ventricular arrhythmias originating from the left ventricular outflow tract, tricuspid and mitral annular regions, ventricular septum, and non-outflow tract sites compared with women (27). However, in the present analysis, sex was not independently associated with PVC presence. Therefore, our findings more strongly support the association between systemic inflammatory status, as reflected by SII, and PVC occurrence rather than a sex-specific effect.

The clinical relevance of the present study should be interpreted within the context of its exploratory and observational design. Although SII was independently associated with both PVC presence and high PVC burden, its standalone discriminatory performance for identifying high PVC burden was modest. The optimal SII cut-off value of 505.6 yielded a sensitivity of 50.9% and a specificity of 66.2%. At this threshold, the positive predictive value was 11.1% and the negative predictive value was 94.2%; however, these predictive values should be interpreted in light of the relatively low prevalence of high PVC burden in the study population. Therefore, the SII cut-off should not be regarded as a definitive diagnostic or clinical decision-making threshold, and SII should not be used as a standalone marker for identifying high PVC burden. Rather, SII may represent an easily obtainable and low-cost inflammatory marker that provides complementary information when interpreted together with clinical evaluation, echocardiographic findings, and rhythm monitoring. These findings support the potential value of SII as an adjunctive research and risk-contextualization marker, while further prospective studies are needed before routine clinical application can be recommended.

## 5 Limitations

This study has several limitations that should be acknowledged. First, because of its retrospective and single-center design, it is hard to apply the results to other situations and figure out a relationship to those situations. Second, the exclusion of patients with structural heart disease limits the generalizability of the findings to wider patient populations, third the lack of long-term follow-up data prevents the assessment of clinical outcomes, including progression to in review cardiomyopathy or heart failure, Fourth, although standard echocardiographic data including LVEF were available and patients with overt structural heart disease or reduced systolic function were excluded, advanced echocardiographic parameters for detecting subclinical cardiac dysfunction, such as global longitudinal strain and detailed diastolic function indices, were not available. Another limitation is that established inflammatory markers such as C-reactive protein and erythrocyte sedimentation rate were not routinely available and therefore could not be compared with SII. Consequently, the present study cannot determine whether SII provides incremental value over conventional inflammatory biomarkers. In addition, due to the retrospective nature of the study, the exact number of all patients who underwent Holter monitoring during the study period and the number excluded because of incomplete laboratory or echocardiographic data could not be reliably determined. Therefore, the findings should not be interpreted as prevalence estimates of frequent PVCs among all patients referred for Holter monitoring. Moreover, the limited sample size in the high PVC burden subgroup may have influenced the statistical power of the results, Finally, potential confounding factors, including lifestyle, medication, and circadian variation, were not controlled, and gender-based hormonal influences were not examined. Despite these limitations, the significant association between SII and both the presence and severity of PVCs in a well-defined group without structural heart disease confirms the idea that systemic immune-inflammation is involved in the presence of ventricular arrhythmias. Prospective studies that include serial SII measurements, comprehensive cardiac imaging, and long-term clinical outcomes are necessary to validate the prognostic and potentially causative significance of these findings.

## 6 Conclusion

Our study demonstrated an association between the Systemic Immune-Inflammation Index (SII) and both the presence and burden of premature ventricular contractions. Higher SII levels were observed in patients with PVCs and in those with greater PVC burden. These findings suggest that SII may serve as a readily available inflammatory marker associated with frequent ventricular ectopy. Further prospective studies are needed to determine the clinical utility of SII and to clarify whether it provides incremental value when combined with clinical evaluation, echocardiographic findings, and rhythm monitoring.

## Data Availability

The original contributions presented in the study are included in the article/Supplementary Material, further inquiries can be directed to the corresponding author.
